# Microfluidic Approaches to Bacterial Biofilm Formation

**DOI:** 10.3390/molecules17089818

**Published:** 2012-08-15

**Authors:** Junghyun Kim, Hee-Deung Park, Seok Chung

**Affiliations:** 1 School of Mechanical Engineering, Korea University, Seoul 136-713, Korea; 2 School of Civil, Environmental and Architectural Engineering, Korea University, Seoul 136-713, Korea

**Keywords:** microfluidics, biofilms, bacteria, bacterial biofilm

## Abstract

Bacterial biofilms—aggregations of bacterial cells and extracellular polymeric substrates (EPS)—are an important subject of research in the fields of biology and medical science. Under aquatic conditions, bacterial cells form biofilms as a mechanism for improving survival and dispersion. In this review, we discuss bacterial biofilm development as a structurally and dynamically complex biological system and propose microfluidic approaches for the study of bacterial biofilms. Biofilms develop through a series of steps as bacteria interact with their environment. Gene expression and environmental conditions, including surface properties, hydrodynamic conditions, quorum sensing signals, and the characteristics of the medium, can have positive or negative influences on bacterial biofilm formation. The influences of each factor and the combined effects of multiple factors may be addressed using microfluidic approaches, which provide a promising means for controlling the hydrodynamic conditions, establishing stable chemical gradients, performing measurement in a high-throughput manner, providing real-time monitoring, and providing *in vivo*-like *in vitro* culture devices. An increased understanding of biofilms derived from microfluidic approaches may be relevant to improving our understanding of the contributions of determinants to bacterial biofilm development.

## 1. Introduction

Aquatic bacteria preferentially adhere to solid surfaces and form bacterial community [1]. Bacterial aggregates form biofilms, which are structured microbial communities supported by an extracellular polymeric substrate (EPS). Biofilm development is not simply a passive aggregation of cells. The biological ecology dynamics involve physical, chemical, and biological interactions with the microenvironment.

The biofilm matrix provides microorganisms with a protective shield that contributes significantly to several clinical challenges, including symptomatic inflammation, antibiotic resistance, recurrence, and the spread of infectious emboli [2,3,4,5,6]. In biofilms, cells can survive under harsh environments, for example, at high temperatures or in the presence of antibiotics. The biofilm matrix improves a microbe’s opportunities for proliferation inside the body. These considerations may explain the correlation between dental hygiene and nosocomial diseases [7,8].

Many studies have examined the effects of genetic and environmental factors on biofilm development, including shear stress [9], surface topography [10], quorum sensing signals [11,12], temperature, and nutrient concentration [13,14]; however, a detailed understanding of the mechanism associated with biofilm development and the compounding effects of each factor have not been well characterized. Under these circumstances, microfluidic approaches present a promising platform for bacterial biofilm research. Microfluidics provide unprecedented control over the flow conditions, accessibility to real-time observation, high-throughput testing, and *in vivo* like biological environments. An understanding of the mechanism underlying biofilm formation and the design of advanced experiments using microfluidic could help identify solutions to biofilm-related problems, such as biofilm infections. In this review, we discuss bacterial biofilm development in terms of the structurally and dynamically complex interactions between the biological system and its environment. Microfluidic approaches provide promising tools, and their use in investigations of the fundamental mechanism of biofilm development will be introduced.

## 2. Biofilm Development

### 2.1. Why Do Planktonic Cells form Biofilms?

What are the advantages of biofilm formation for the survival and proliferation of bacteria? Several factors contribute to the success of biofilm-forming bacteria [15,16]. The biofilm matrix shields the microbes from harsh external conditions, such as shear stress, nutrient deprivation, pH changes, oxygen radicals, disinfectants, and antibiotics. Bacterial cells within biofilms can withstand these stressful conditions because the EPS neutralize or bind antimicrobial agents, thereby protecting cells from physical stress [17]. Delay on cell maturation in the biofilm matrix and induction of *rpoS*-mediated stress response could cause resistance to antibiotics [18]. Adhesion to surfaces and biofilm formation provide favorable habitats for cells. Biofilms offer a degree of stability and function catalytically in the sense that cells remain localized in close proximity. The microbial communities in biofilms consist of a variety of cells and tend to behave as multicellular organisms. The bacterial cell types cooperatively survive as a whole community [19,20].

### 2.2. Biofilm Development Sequence

**Figure 1:**
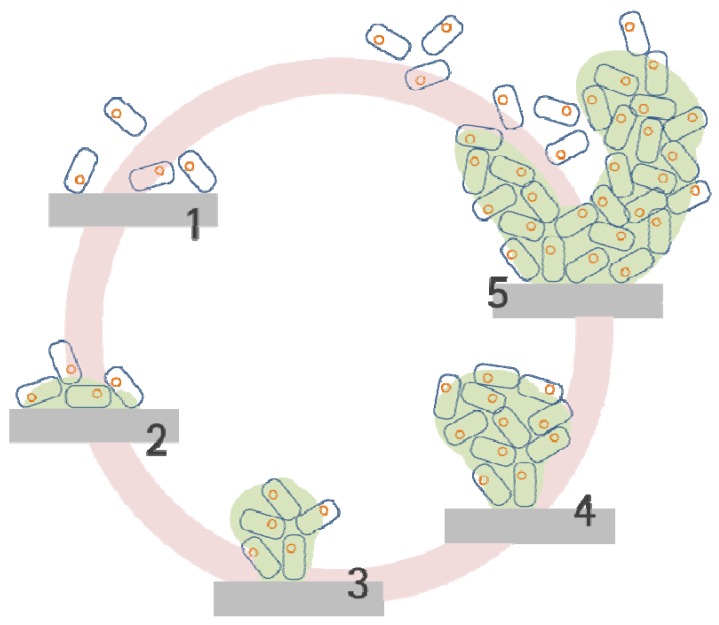
*Pseudomonas aeruginosa* biofilm development sequence; step 1 initial adhesion of a bacterial cell to a surface; step 2 induce irreversible adhesion by EPS generation; step 3 early structural development; step 4 maturation of the biofilm; step 5 dispersion of cells from the biofilm matrix.

Studies have revealed that bacterial biofilm development by *Pseudomonas aeruginosa* proceeds through a series of steps (Figure 1) [21]. The initial two steps are characterized by the loose adhesion of planktonic cells to a surface and the production of EPS. Planktonic bacterial cells can approach surfaces under bacterial motility, or under physical forces, such as Brownian motion, van der Waals attraction forces, gravitational forces, surface electrostatic charge, and hydrophobic interactions. As bacterial cells approach other cells or come within 50 nm of a surface, specific interactions between the two entities become significant. The interactions are a direct function of the free energy characteristics of and distance between the two entities [22]. Steps three and four of biofilm development entail the cellular aggregation and the subsequent growth and maturation processes. Depending on the nutrient conditions, biofilm structures can be slab or mushroom-like in shape [23]. Step five involves the dispersion of single cells from the biofilm matrix and film detachment by erosion [24,25]. Although several studies have investigated the initial steps of biofilm formation, more intensive investigations of biofilm detachment, which is a major cause of biofilm-related disease, would be valuable and inform therapeutic strategies.

### 2.3. Determinants of Biofilm Development

Biofilms have been studied by many researchers, and a variety of determinants of biofilm development have been revealed. Biofilm formation is influenced by both gene expression and environmental conditions, including surface properties, shear stress, quorum sensing signals, and the characteristics of the aqueous medium. These factors are not the only important considerations, but they provide significant contributions to biofilm development. The most dominant factor and the magnitude of the relative contributions of other factors remain under debate among biofilm researchers.

#### 2.3.1. Gene Expression

Molecular techniques, such as random transposon mutagenesis and knockout mutant studies, have revealed that biofilm-specific gene expression is involved in biofilm formation [16]. Jefferson *et al*. organized the knowledge of bacterial genes and their functionalities in the context of biofilm development [15]. In the case of *P. aeruginosa*, *crc* regulates global carbon metabolism, *algC* promotes alginate synthesis, and *PA2128* reduces biofilm development by producing a probable fimbrial protein [26,27,28]. Although many studies have revealed biofilm-specific genes and their effects, genetic-based approaches have their limits [29,30,31,32,33,34,35,36,37,38,39,40,41,42,43,44,45,46]. These studies compare biofilm formation by mutant and wild-type strains in high-throughput micro wells. Such studies limit the examination of biofilm development to particular stable conditions with low shear flow and no nutrient exchange. Comparative biofilm growth studies are assessed within short periods of time and consider only the early stages of biofilm development. These limitations may be compensated by revealing interaction between gene expression and environmental conditions.

#### 2.3.2. Surface Properties

Surface properties, including the surface composition, morphology, structure, and material properties, affect biofilm development from the earliest stages of adhesion to the final stages of dispersion. Studies have shown that biofilm formation and colonization is promoted by rough surfaces due to the presence of beneficial local environments and the increased opportunities for biofilm formation. For example, the pockets in rough surfaces provide a protective habitat with reduced shear stress [47]. Bacterial cells more tend to adhere more strongly to hydrophobic and non-polar surfaces than to hydrophilic surfaces [48]. Additionally, porous materials are associated with a higher degree of biofilm formation compared with dense and smooth materials. The attachment of bacterial cells to porous substrates is affected by the degree of porosity, the pore size, and the permeability distribution [49,50].

After bacterial cells adhere to a surface, the biofilm matrix is influenced by the architecture and electronic properties of the solid surface. Cations on a surface, such as magnesium and calcium, actively contribute to biofilm cohesion and development. They act as lipopolysaccharide cross-linkers by promoting the integrity of the outer cell membrane [51]. Modified titanium surfaces were used to exam the effects of the solid surface tension on biofilm adhesion and cohesion forces. *Pseudomonas fluorescens* biofilms endured and thrived under a high shear stress when cultured over a chloropropyl-terminated surface, whereas biofilm formation was less extensive on an alkyl-terminated surface [52]. The surface architecture of the abiotic target was demonstrated to affect the metabolism and morphology of the colonizer. Nanometer sized topographical features on titanium surfaces reduced the bacterial adhesion forces and promoted selected target cells (e.g., osteoblasts). The size and shape of the nanostructure either positively or negatively influenced biofilm formation [10].

#### 2.3.3. Hydrodynamic Conditions

Hydrodynamic conditions significantly influence biofilm development [53]; whether shear stress, for example, enhances or hinders biofilm development remains under debate. During the initial stages of biofilm formation, shear stress can increase the residence time of the bacterial cells at the interface, providing more opportunities for bacterial cells to adhere and disperse [24]; however, many experimental studies have reported that shear stress acts as an inhibitor of biofilm development. Bacterial cells subject to high shear stress tend to form thin monolayer biofilms [54,55]. Shear stress can slow down maturation, maintaining biofilms in a “young” or early stage, and decrease bacterial diversity in a biofilm [9].

Under low shear stress conditions, biofilms develop thick multilayer structures. These structural adaptations can affect the bacterial susceptibility to antibiotics. Under high shear stress conditions, the viability of biofilms to gentamicin was observed to decrease. Two explanations for this effect were presented: high flow rates promote molecular diffusion, and thin biofilm structures easily establish a significant antibiotic gradient through their matrix [54]. Additionally, the time-dependent stress profile influenced the success of initial biofilm colonization. For a given mean shear stress, non-uniform shear stress more effectively prevented biofilm formation than a uniform stress distribution [25]. Although the shear stress interrupted biofilm formation and covered a large space, it assisted with the clumping and dispersion of the biofilms. Under a high flow rate, the biofilm area and thickness was reduced, but the total number of bacterial cells in the fluid was high [56]. Clumped biofilm debris migrated with the flow and provided opportunities for colonizing new niches [16].

Hydrodynamic conditions can have contradictory influences on biofilm development. Shear stress suppresses the development of a biofilm matrix but provides more opportunities for new biofilm formation by increasing the residence time and improving motility. A balance between the two opposing effects may be struck to provide optimal shear stress conditions for promoting biofilm growth.

#### 2.3.4. Quorum Sensing Signals

Quorum sensing is the regulation of gene expression in response to changes in the cell population density. Bacterial cells produce and release chemical signaling molecules called autoinducers, the concentration of which increases as a function of the cell density [57]. For example, *Pseudomonas aeruginosa* cells require *lasI* to develop mature biofilms. *lasI-*Mutant cells were observed to terminate biofilm formation at the flat structure, micro-colony state, rather than forming mature biofilm colonies that has mushroom-like shape [58]. Other studies have shown that the induction of quorum sensing is related to a critical biofilm depth. Once biofilm thickness exceeds a critical depth, quorum sensing is induced. The value of the critical depth varies with the pH of the surrounding fluid [59]. Biofilms structures are mainly composed of EPS. In almost all bacterial biofilms, the biosynthesis of EPS is a quorum sensing-dependent process involving auto-inducer molecules. In Gram-negative bacteria, *N*-acyl-L-homoserine lactone (AHL) autoinducers mediate quorum sensing and biofilm formation. The AHL autoinducer type depends on the bacterial species. Dickschat provided a summary and categorization of all known AHL autoinducers produced by bacterial species [11].

#### 2.3.5. Characteristics of the Aqueous Medium

Autoinducers do not act independently from other influence factors, such as nutrient concentration, pH, or the concentration of carbon dioxide or oxygen. The role of quorum sensing in *Pseudomonas aeruginosa* biofilm formation depends on the nutritional conditions. Depending on the nutrient sources, quorum sensing can regulate or not regulate the swarming motility of the bacterial cells, thereby influencing the biofilm structure. Cells with a low motility form aggregates, leading to more structured biofilms, whereas cells with a high motility spread across a surface, leading to flat biofilms [60]. Hunt *et al.* proposed a hypothesis governing the role of nutrient starvation in biofilm detachment. Under a nutrient-starved environment, biofilm erosion detachment occurs under homogenous hydrodynamic conditions. Computer models of biofilm dynamics suggested a starvation-dependent detachment mechanism. Insufficient nutrition causes void areas toward the centers of biofilm colonies that induce biofilm sloughing detachment [61].

Oxygen and carbon dioxide are important components and determinants of biofilm development. The concentration of dissolved oxygen influences the initial attachment of cells and EPS production [13,62]. Experimental results showed that the adhesion of *Pseudomonas aeruginosa* to a substrate changed with the oxygen gradient. Planktonic cells prefer to adhere to surfaces in locations that include higher levels of dissolved oxygen [13]. A comparison of the effects of carbon and oxygen concentration showed that oxygen-limited biofilms contain more extracellular polymer carbon than carbon-limited biofilms. High extracellular polymer carbon enhances the structural strength of a biofilm matrix and decreases erosion detachment under shear stress [62]. The concentration of carbon in the fluid determines the proportion of extracellular polymer carbon in the biofilm matrix, which directly affects the susceptibility of a biofilm to shear stress. Dense phase carbon dioxide (DPCD) was shown to inactivate biofilms that were completely wet but not immersed in water. DPCD is one of the most promising techniques available for controlling microorganisms that display a high antimicrobial resistance [63]. Table 1 summarizes the effects of the environmental conditions, including the surface properties, hydrodynamic conditions, quorum sensing signals, and characteristics of the medium.

**Table 1:** Effects of the environmental conditions on biofilm development.

Environmental conditions	Effect on biofilms	Species	Reference
**Surface properties**			
surface roughness	Positive	*P. aeruginosa*	[47]
hydrophobicity	Positive	*Pseudomonas* sp.	[47]
non-polar surface	Positive	*Pseudomonas* sp.	[48]
porosity	Positive	*Corynebacterium*, *Rhodococcus*, *Gordona*	[49,50]
cations on the surface	Positive	*P. fluorescens*	[51]
chloropropyl-terminated surface	Positive	*P. fluorescens*	[52]
alkyl-terminated surface	Negative	*P. fluorescens*	[52]
nanostructure of the surface	Positive/Negative	*S. aureus*, *S. epidermidis*, *P. aeruginosa*	[10]
**Hydrodynamic conditions**			
residence time	Positive	*P. aeruginosa*	[24]
shear stress at the interface	Positive/Negative	*P. aeruginosa*, *P. fluorescens*	[54,55,56]
hetero-stress distribution at the interface	Negative	*P. aeruginosa*	[25]
**Quorum sensing signals**			
quorum sensing autoinducers	Positive	*Gram-negative bacteria*	[57]
**Characteristics of the aqueous medium**			
nutrient source	Positive/Negative	*P. aeruginosa*	[60]
nutrient starvation	Negative	*P. aeruginosa*	[61]
oxygen concentration in the fluid	Positive	*P. aeruginosa*	[13]
carbon dioxide concentration in the fluid	Positive	*P. putida*	[62]
dense phase carbon dioxide	Negative	*P. aeruginosa*	[63]

## 3. Microfluidic Approach

### 3.1. Advantages of Microfluidics

**Figure 2:**
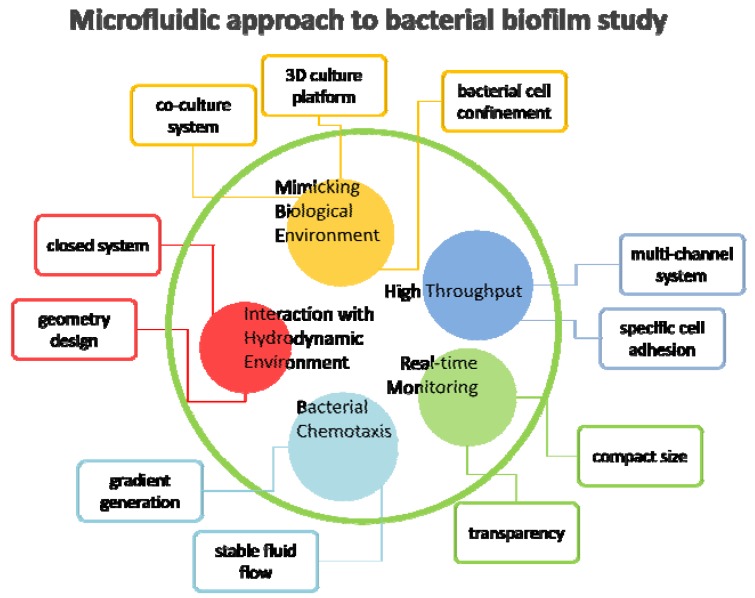
Advantages of microfluidics approach to bacterial biofilm studies. Microfluidics and micro-fabricated platforms have various characteristics as shown in the box that are suitable for biofilm studies. These characteristics allow developing micro-platforms for evaluating the interaction with hydrodynamic environment and bacterial chemotaxis, high throughput biofilm array, real-time monitoring, and *in vivo* like biological environments.

Microfluidic devices manipulate fluids that generally constrained to a small environment, sub-millimeter scale. They are easily fabricated of wafers, plastics, elastomers, papers, and glass. Many studies have applied microfluidic technology due to its remarkable potentials; small liquid volume control, confining cells and molecules in a spatial geometry, temperature control and precise gradient generation, enabling low cost, rapid and precise analysis. The microfluidic devices present a promising platform for bacterial biofilm studies (Figure 2). They provide closed system where bacterial biofilms could interact with hydrodynamic environments. It allows developing mathematical models that account influences of these interactions and revealing the effects of hydrodynamic conditions (e.g., shear stress) on development of biofilms [64]. Fluid flows in these devices are very stable and yield fast response times due to the low Reynolds number, to generate gradient of chemical attractant and monitor bacterial chemotaxis. Their compactness and transparency permit observation of biofilm development in real time using high-throughput arrays. The short diffusion time and small scale facilitate bacteria culture and biofilm formation because it is easy to set up a variety of favorable conditions. The environments in microfluidic devices can be used to create models of the *in vivo* conditions in 3D culture platforms. These features were beneficial not only for assessing the contributions of each influencing factor to biofilm growth, but also for revealing the compounded effects. Microfluidic approach can potentially reveal the mechanism underlying biofilm formation and resolve a number of biofilm-related problems.

### 3.2. Microfluidics Approaches in Bacterial Biofilm Studies

#### 3.2.1. Interaction with Hydrodynamic Environment

**Figure 3:**
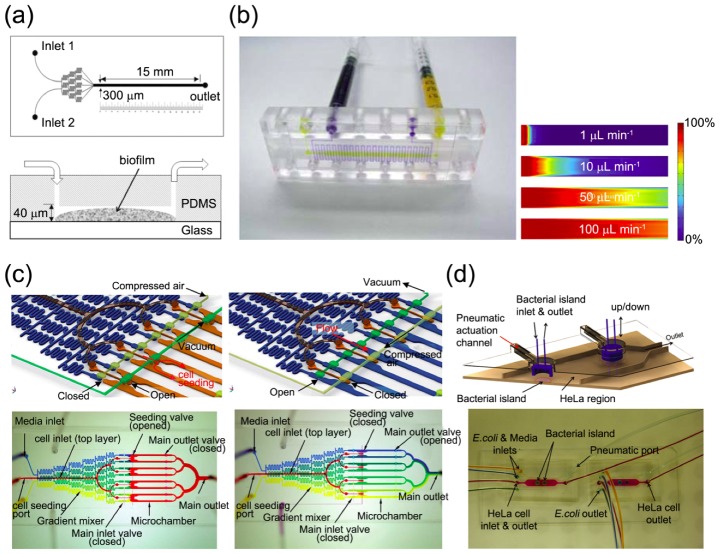
Microfluidic devices used in the bacterial biofilm studies. (**a**) Schematic diagram of a microfluidic device used for bacterial biofilm formation. The effects of shear stress were quantified by analyzing the biofilm area in the microfluidic channel [66]; (**b**) Multi-layer microfluidic device for generating an oxygen gradient. Blue dye was injected into the channel and yellow dye was injected into the chamber. Simulation results modeled the oxygen saturation gradient in the growth chamber [13]; (**c**) Microfluidic flow cell for high throughput bacterial biofilm studies. The device included a glass coverslip and two PDMS layers. A bacterial biofilm developed in the microfluidic channel upon exposure to the signaling molecules [75]; (**d**) Model for the co-culture of epithelial cells and bacterial biofilms. A 3D rendering image showed the pneumatically-actuated trapping regions for producing biofilm islands among the epithelial cells. The colored dyes show the different regions of the co-culture device [78].

Microfluidic approaches enable us to access the effects of the hydrodynamic conditions, such as the shear stress, antibiotics under flow conditions, and stress distributions. *Polydimethylsiloxane* (PDMS) chips are used to prepare micro-channels that permit control over the hydrodynamic conditions under which bacterial cells are cultured. Bahar *et al.* used microfluidic flow chambers to assess bacterial adhesion of *acidovorax citrulli* [65]. Lee *et al.* characterized the structural changes displayed by biofilms under shear stress imposed in a PDMS microfluidic device with a simple straight channel [55]. Shear stress negatively impacts biofilm development in mature biofilms or during the dispersion stages; however, during the initial and adhesion stages, shear stress promotes biofilm formation by offering more nutrient and opportunity for dispersion [66] (Figure 3a). In the absence of stress and the presence of conditions favorable for bacterial cell growth, biofilms form slowly relative to biofilm formation under stressful conditions. The flow channel geometry may be used to modulate the distribution of shear stress on a biofilm interface [54]. At different locations in a channel, bacteria cells can experience different degrees of shear stress, which changes the biofilm coverage, thickness, and viability. Microfluidic channels may be designed to elucidate the combined effects of several influencing factors on biofilm formation. Multi-channel devices have been used to assess the effects of a poly-hydrolyzing enzyme (dispersin B) and/or an antibiotic (rifampicin) on biofilm detachment. Dispersin B and rifampicin treatment induced the removal of most biofilms; however, at the corner, the biofilm remained intact due to an insufficient shear flow. The combined effects of the hydrodynamics and antibiotics provide an effective tool for biofilm removal [55]. With these experimental works, microfluidic approach could be valuable way to developing mathematical models. Janakiraman *et al.* introduced a mathematical model based on biofilm growth in a closed system, where biofilm development and hydrodynamic environments are interlinked [64]. To verify prediction of model, they used microfluidic chambers as closed system. Based on interaction among biofilm development, mass transport, and hydrodynamics the results of model was well matched with experimental results. When bacterial cells develop biofilms, they continuously interact with their environment and this interaction finally influence to growth of biofilms. Providing closed environment, microfluidic approach allow researchers to understanding a biofilm as a colony of live cells that altered continuously with its environments.

#### 3.2.2. Bacterial Chemotaxis

Stable flow conditions in microfluidic devices facilitate the generation of flow-free, steady gradients of arbitrary shape. The chemotaxis of free-swimming or surface-adhered bacterial cells plays a fundamental role in biofilm formation and dispersion. Many examples of gradient generation in microfluidic channels for the purpose of bacterial chemotaxis study rely on flow conditions. Parallel flow devices called T-sensors operate based on the confluence of three streams, which join into a single micro-channel [67,68,69]. The distribution of bacteria is then measured at the end of the channel, yielding a cumulative response to the evolving nonlinear gradient experienced along the micro-channel [70]. Flow-free chemotaxis generators set up a gradient based on molecular diffusion only, and no flow is present. In this case, a flow structure is used to set up an initial gradient. The flow is then turned off, thereby allowing the gradient to evolve by diffusion alone [71,72,73]. When operated on a short timescale, the gradient in this type of device can be approximated as being steady-state. Such gradients are useful for quantifying chemotaxis if the timescale of the gradient relaxation is much longer than the characteristic times of the sensing and behavioral processes [70]. The incorporation of porous materials, such as hydrogels or porous membranes, in microfluidic devices has enabled the creation of steady chemical gradients in an environment completely free of flow or shear [71]. Skolimowski *et al.* showed that the attachment of *Pseudomonas aeruginosa* to a substrate varied depending on the oxygen concentration, which was modulated using a gas-permeable membrane (Figure 3b) [13].

#### 3.2.3. High-Throughput Analysis

The compactness of microfluidic devices makes them suitable for high-throughput arrays and *in situ* monitoring. Eun *et al.* examined arrays of biofilm islands with a variety of shapes using thin polymer stencils as scaffolds [74]. PDMS stencils induce stable biofilm formation at a desired position. The diversity of the biofilm shapes makes it possible to address the effects of colony geometry on the organization, physiology, and homeostasis of biofilms. Kim *et al.* developed a PDMS-based microfluidic flow cell device with a dual-layer structure for investigating biofilm formation and organization in response to different concentrations of soluble signals (Figure 3c) [75]. It revealed biofilm formation in response to soluble gradients of chemical signals (e.g., 7-hydroxyindole and isatin). It could also be used to screen antibiotics and biofilm inhibitory cocktails. Benoit *et al.* developed a microfluidic device for the high-throughput screening of biofilm viability under flow on 96 individual biofilm islands [76]. Peng Sun *et al.* revealed the effect of loading density of bacterial cells on biofilm formation using long-term culture arrays. They evaluated antibiotics under static (no-flow) condition [77], and found that the growth rate of biofilm was constant regardless of initial loading density when bacterial cells entered long-phase proliferation. It is important to maintain uniform bacterial cell density in microfluidic based high-throughput arrays. From simple arrays to multi-chemotaxis generators, microfluidic technology has offered a first step toward investigating the compound effects of physical, chemical, and biological factors on biofilm formation in high-throughput.

#### 3.2.4. Real-Time Monitoring

Microfluidic devices may be combined with existing quantification tools, such as confocal and fluorescence microscopy, to enable real-time monitoring of biofilm developments [79,80] (Table 2). In this application, microfluidic device was used as a flow-cell with precise controllability in compact space. Holman *et al.* introduced an open-channel microfluidic system for the *in situ* chemical imaging of bacterial activity in biofilms using synchrotron radiation-based Fourier transform infrared (SR-FTIR) spectroscopy. This system directly monitored the bacterial activity and biochemistry at a molecular level within a biofilm over a long period of time [81]. Meyer *et al.* designed a microfluidic platform that enabled the simple optical monitoring of bacterial biofilms. Biofilm formation could be monitored during growth by measuring the changes in the optical density or electrical residence using off-the-shelf electrical components [82]. Richter *et al.* developed a biochip for the online monitoring of biofilm dynamics. This system used high-density integrated capacitors for non-invasive measurements. Biofilm development in the channel induced changes in the voltage at the capacitors, yielding electrical signals [83]. These works made possible that researchers monitored biofilm developments in real-time, only with simple microchannel as a flow-cell, remaining much to be improved with complicated microfluidic circuits.

**Table 2:** Real-time methods to monitor biofilm development.

Analysis techniques	Microfluidic approach	Acquired information	Reference
Fluorescence microscopy	*generating chemical (antibiotic) gradient*	antibiotic susceptibility of bacterial biofilms	[79]
Confocal reflection microscopy	*micro scale culture chamber*	biofilm growth with time	[80]
SR-FTIR spectroscopy	*circumventing water-absorption barrier*	molecular level within biofilms over a long timebiomolecule synthesis during biofilm development	[81]
Optical density(LED array & photodiodes)	*transparent culture chamber*	change in biofilm optical density over the growth period	[82]
High-density interdigitated capacitors (µIDES)	*dielectric micro-sensors integrated transparent biochip*	changes of optical density and impedance caused by biofilm growthdynamic responses of biofilms to shear stress and antimicrobial agent concentration	[83]

#### 3.2.5. Mimicking Biological Environments

In the environment or, for example, the human body, bacterial cells are exposed to diverse environmental conditions and must react in order to survive. Microfluidics and micro-fabricated tools make it possible to produce biofilm culture platforms that mimic the *in vivo* conditions experienced by bacterial cells. In the buccal cavity, *Streptococcus mutans* is the primary etiological agent responsible for dental caries. Using a microfluidic device with glass beads, Shumi *et al*. simulated the interproximal space of teeth [84]. They quantified the effects of sucrose and metal ions on biofilm formation in the gaps between glass beads. In the human gastrointestinal (G1) tract, intestinal epithelial cells and non-pathogenic bacteria co-exist as a form of biofilm. In the event that pathogens invade the G1 tract, the biofilm equilibrium becomes perturbed, and commensal bacterial cells from the biofilm matrix navigate toward the pathogens. This is a key step in the infection processes. Microfluidic co-culture models enable the independent culturing of epithelial cells and bacterial biofilms in an effort to exam the role of the commensal microenvironment in pathogen colonization. A pneumatically-actuated system was used to form reversible islands that allow for the development of bacterial biofilms along with epithelial cell monolayer culturing (Figure 3d) [78].

As three-dimensional culture systems, microfluidic approaches provide a more *in vivo*-like environment for bacterial cells in *in vitro* experiments. Lee *et al*. examined the connection between biofilms and infection mechanisms in orthopedic implants using a three-dimensional culture device. A multi-channel microfluidic device was used to observe the development of osteoblasts in three-dimensional tissue-like structures. This study revealed how osteoblast development was influenced by the phenotype of the *Staphylococcus epidermidis* [85]. Planktonic cells forming biofilms communicate with one another using quorum sensing signals. Micro-encapsulation technologies may be used to study the quorum sensing mechanisms and growth of bacterial biofilms by confining one to three cells in microcapsules. A few cells confined in a very small volume results in the accumulation of auto-inducers, which induce quorum sensing growth. This strategy demonstrated that quorum sensing is a function of the measure of biomass per unit volume [86].

## 4. Conclusions

Bacterial biofilms are highly dynamic and sensitive to their environments, which make their analysis and control more challenging. Biomedical and bioengineering research studies of the various determinants of biofilm formation have been conducted, from studying the genetic expression patterns to the environmental conditions; however, little is known about the relative contributions of the genetic and environmental factors. A single-species bacterial biofilm had been well studied in various ways however studies about multi-species bacterial biofilms still stay at the beginning stage, remaining a lot of unknowns in bacterial communication. Biofilm engineering like biofilm catalysts and array is in trouble due to uncontrollable characteristics of bacteria. Understanding of microfluidic approach in bacterial biofilm studies could help researches to overcome these hurdles and solve the problems. The improved analyzing ability makes it possible to reveal compounded effects of genetic and environment factors. Lab-on-a-chip system could allow to culture multi-species biofilms without confusing and misunderstanding. Highly improved controllability of microfluidics helps to regulate biofilm growth in catalysts and high-throughput arrays. The knowledge gained from applying microfluidic approaches has significantly improved our understanding of biofilm formation and function.
